# Improving healthcare value: Lessons learned from the first decade of *Choosing Wisely*®

**DOI:** 10.1002/jhm.12969

**Published:** 2022-10-03

**Authors:** Wendy Levinson, Jerome A. Leis

**Affiliations:** ^1^ Department of Medicine University of Toronto Toronto Ontario Canada; ^2^ Division of Infectious Diseases, Sunnybrook Health Sciences Centre Toronto Ontario Canada; ^3^ Centre for Quality Improvement and Patient Safety University of Toronto Toronto Ontario Canada

Low‐value care threatened the sustainability of healthcare even before the COVID‐19 pandemic with up to 30% of care estimated to be wasteful—meaning it does not add value and may even be harmful to patients.^1^ The direct costs of COVID‐19, along with backlogs of delayed care, and staffing shortages, have only intensified fiscal constraints. Now more than ever, healthcare systems must find ways to better utilize finite healthcare resources if they are to be sustainable.

It was one decade ago that *Choosing Wisely*® began in the United States to reduce the use of unnecessary medical tests, treatments, and procedures (i.e., low‐value care), and has since spread to over 30 countries. To date, it has been primarily a bottom‐up campaign. The lists by professional societies were a starting point that emphasized many of the “easy wins” to reducing low‐value care, but they are by no means the largest causes of waste in the healthcare system. Most unnecessary care is intertwined with appropriate care and is much more challenging to “de‐implement.”^2^ But in some countries, like Canada, *Choosing Wisely*® has moved from an awareness campaign to focus on system re‐design.

Not surprisingly, the evidence shows that the campaign has had modest successes in reducing low‐value care, typically at single sites and involving relatively easy‐to‐eliminate tests or treatments.^3^ Table 1 lists examples of strategies shown to reduce low‐value care that can be implemented across the hierarchy of intervention effectiveness.^4^ This framework is a useful way to conceptualize quality improvement interventions where the lowest ranking is the most feasible but least effective as compared to the higher levels that are increasingly effective yet the hardest to implement. Education and training, which represents the majority of where *Choosing Wisely*® has focused to date, are at the bottom of the hierarchy because although necessary, these are rarely sufficient in order to change longstanding practice patterns, particularly because many drivers of overuse are unrelated to a gap in knowledge.

**Table 1:** Examples of proven strategies for reducing overuse of healthcare resources and their limitations

Level of hierarchy of intervention effectiveness	Examples	Strengths and limitations
Education and training	Physician Education and awareness campaigns; Patient education at point of care; Shared decision making	Good for galvanizing activity, but generally will not achieve concrete results on its own
	Audit‐and‐feedback (e.g., performance report cards)	Small effect sizes in most cases and intrinsic limit on the number of concurrent interventions appealing to professional pride and requiring change strategies for each target
	Dedicated programs for overseeing appropriate use of services	Some robust successes (e.g., stewardship programs for antibiotics, blood products) but limited number of targets where we can afford to have such intensive oversight; Hard to apply to problems that cross silos of care (e.g., chronic opiates, overuse of antipsychotics and sedatives in the elderly)
Rules and policies	Removing routinely ordered low‐value investigations from order sets; Limiting number of days for ‘Daily bloodwork'; Decoupling investigations (e.g., PT/INR, ALT/AST, urea/creatinine)	Limited number of targets; True cost savings usually much smaller than generally appreciated
	Vetting orders (e.g., for advanced imaging) using appropriateness criteria	Difficulty of designing a robust intervention without interfering with workflow for frequently ordered tests
Reminders, checklists, and double checks	Appropriateness criteria applied before testing/procedures	Resource intensive but can be successful in reducing overuse
Simplification and standardization	Using data demonstrating regional variation in practice patterns to look for opportunities to standardize practice; Bundled payments for care improvement can drive simplification and standardization)	Substantial resistance from stakeholders rewarded by historic approach; Concerns about unintended consequences (e.g., increased wait times once providers not incentivized by volume)
Automation and computerization	Decision support embedded in clinical information systems	Small effect sizes in most cases and the problem of alert fatigue limits the number of concurrent applications possible
Forcing functions	De‐funding specific low‐value services (eg. Vitamin D testing)	Highly effective but too few tests and treatments have such infrequent legitimate indications that they can be successfully defunded

Dedicated stewardship programs are a successful scalable example for reducing unnecessary antimicrobial use because they do not focus on education alone but additionally provide other interventions such as real‐time feedback on prescribing practices.^5^, ^6^ This model can be applied to other targets (e.g., antithrombotic agents, psychoactive drugs in the elderly) but it is not feasible to dedicate personnel to oversee stewardship programs for every overuse problem. Similarly, we cannot burden clinicians with concurrent performance report cards for more than a handful of targets.

Overuse is baked into many of our existing processes of care. Simple changes can be implemented at the level of organizational rules and policies, such as revising order sets and other process redesigns to reduce redundant or unnecessary laboratory and radiologic investigations.^7^ Yet, the underappreciated distinction between fixed and variable costs means these process changes, often initiated to save money, generate only the illusion of substantial savings.^8^

Double‐checks and reminders such as preprocedural screening of patients for appropriateness, for example, prior to knee and hip surgery, are often effective but resource intensive.^9^ Addressing regional variations in practice and providing feedback to facilities or health regions may help to inform opportunities but making improvements at this broader system level is unlikely to occur without strong alignment with physician leadership. The results of bundled payment initiatives to promote standardization and simplification have been underwhelming, in part due to this inability to influence care provided across the spectrum of care.^10^

Although effective, automated interventions such as computerized decision support typically produce small gains—absolute improvements on the order of 5%, whether for increases in recommended care or reductions in low‐value care.^11^ Unfortunately, we cannot implement more than a handful of such interventions at any given time due to the impact on clinicians. Excessive use of electronic reminders has resulted in clinicians overriding such alerts virtually all the time.^12^

Finally, defunding low‐value services altogether is an example of a forcing function, which ranks at the top of the hierarchy of effectiveness, where the test or treatment is rendered unavailable due to insufficient appropriate indications. Making Vitamin D levels an uninsured test quickly resulted in an over 90% decrease in ordering.^13^ Unfortunately, too few tests and treatments have such infrequent legitimate indications that they can be successfully defunded.

## NEXT STEPS

We believe that implementing more of the same kind of strategies is unlikely to produce significantly better value in our healthcare system. More substantive reductions in low‐value care will depend on a new approach—one that tackles broader system‐wide targets through an approach that combines grass root efforts with health policy changes to healthcare delivery. Such top‐down approaches have been met with resistance in the past because they smacked of rationing—but a decade into the *Choosing Wisely*® era, there is wider recognition regarding the harms of unnecessary care. Creating more efficient care improves quality and safety and is essential to the sustainability of our health care system—a goal physicians and policymakers share.

Too few resource stewardship initiatives have utilized higher levers to drive broader impact because physicians are not aligned with the policy makers or vice versa. Surely the COVID pandemic has taught us that such nimble coordination is possible when a burning platform exists. We offer one illustrative Canadian example applied to a resource stewardship target. A national program designed to reduce the use of red blood cell transfusion was launched by *Choosing Wisely*® Canada to address the nearly 30% of unnecessary transfusions that waste this precious resource and lead to preventable patient complications.^14^ “Using Blood Wisely” was developed based on recommendations by multiple national societies which led to the creation of evidence‐based benchmarks for appropriate use of red blood cell transfusions that could be measured by hospitals.^15^ Accreditation Canada, which sets standards and accredits the majority of hospitals in the country, encouraged hospital leaders to participate and indicated that participating hospitals would receive credit in the required quality improvement component of accreditation. The program was launched during the early phase of the COVID pandemic when donations of blood were very low, and a shortage of blood was a major concern. To date over 220 hospitals accounting for 75% of red blood cell transfusions across Canada are currently participating through encouragement and recognition as a “Using Blood Wisely” hospital. Hospital engagement is high without using a “stick approach” of making participation mandatory.

A key approach to reducing low‐value care in the next decade will be to combine the strengths of bottom‐up, physician‐led initiatives, and top‐down system levers to create change. Policymakers can influence funding, accreditation, or licensure/certification but lack the clinical expertise to judge the appropriateness and determine measures of low‐value care. Furthermore, policymakers are seen as intruding on clinical decision‐making if they set standards without physician leadership. On the other hand, physicians can judge the appropriateness and set benchmarks and facilitate communication with their peers about best practices but lack broader systems to influence change. A combination of approaches is required to increase the impact of the *Choosing Wisely*® campaign.

What strategies can be used to integrate bottom‐up and top‐down to improve healthcare value? First, the target must be an area with broad support for de‐implementation by clinicians. In the “Using Blood Wisely” example, seven national societies had previously listed recommendations regarding the need for judicious use of red blood cells. The same success would not have been achieved without strong consensus by physicians. Second, improvement efforts must focus on changes that support practice change. *Choosing Wisely*® Canada coached physician hospital leaders to implement changes in transfusion practices including adoption of standardized guidelines and real‐time screening of orders by blood bank technologists.^14^ Intervention tools were available and group webinars provided support.^15^ Third, a health system partner(s) must be engaged that will support implementation. In this case, Canadian Blood Services could provide utilization data which they collected regularly. Through their network, leaders of blood services in each of the provinces were identified and participated in setting the national benchmarks, hence gaining their engagement in the process. Fourth, policymakers must be identified who can provide leverage for participation. In this example, the Chief Executive Officer (CEO) of Accreditation Canada sent a joint letter with the Chair of *Choosing Wisely*® to the CEOs of Canadian hospitals. Although the high level of hospital engagement in “Using Blood Wisely” did not require it, a payer could tie benchmarks established by the physicians to funding.

In 2012, *Choosing Wisely*® helped to initiate conversation between physicians and patients about unnecessary and often overused tests and treatment—a challenging and novel conversation at the time. A key lesson from the past decade is that this is not enough. Now we need physicians and policymakers to combine efforts to reduce overuse to improve care and make our health system sustainable. As we emerge from the COVID pandemic, we must transform care to deliver value and build a healthcare system that truly meets the needs of the public for the future.

## CONFLICT OF INTEREST

Jerome Leis and Wendy Levinson receive support from *Choosing Wisely*® Canada.
